# Mechanism of the influence of cationic moieties on the performance of fluid loss additives for well cementing *via* fluorescence labeling

**DOI:** 10.1039/d6ra00459h

**Published:** 2026-03-06

**Authors:** Qi Feng, Chengwen Wang, Tao Yang, Jingwei Yang, Jia Wang

**Affiliations:** a National Key Laboratory of Deep Oil and Gas, China University of Petroleum (East China) Qingdao 266580 China wangcw@upc.edu.cn; b School of Petroleum and Engineering, China University of Petroleum Qingdao 266580 Shandong China; c Petroleum Engineering Research Institute, Dagang Oilfield Company, CNPC Tianjin 300280 China; d Offshore Oil Engineering (Qingdao) Co., Ltd Qingdao 266520 China; e PetroChina Changqing Oilfield Company Xi'an 710000 China

## Abstract

Despite the increasing incorporation of cationic moieties into fluid loss additives for well cementing, their specific adsorption behaviors remain underexplored. Furthermore, precise quantification of adsorption and the elucidation of dispersion mechanisms within cement slurries are frequently compromised by interference from co-existing admixtures, notably retarders. To address these challenges, this study reports the synthesis of a novel fluorescent cationic fluid loss additive (PDDN) *via* precipitation polymerization, utilizing *N*-vinylcarbazole (NVC) as a fluorescent marker. The polymer structure was characterized using NMR and FTIR, while adsorption mechanisms were investigated *via* fluorescence spectroscopy, zeta potential analysis, and high-magnification microscopy. Structural analysis confirms that NVC units are effectively isolated by polymer segments, imparting distinct fluorescence (excitation: 290 nm; emission: 347 nm) without compromising fluid loss control. A highly sensitive standard curve (*y* = 2.7E6*x* + 735) facilitated precise quantification. Notably, this fluorescence labeling technique exhibited superior anti-interference capabilities compared to traditional Total Organic Carbon (TOC) analysis, enabling accurate measurement even amidst high retarder concentrations. Performance evaluations indicate that cationic incorporation enhances slurry rheology and fluid loss performance with minimal impact on compressive strength, though dispersion efficiency is marginally inferior to anionic alternatives. Further comprehensive analysis combining fluorescence, zeta potential, and particle size data reveals that while cationic groups promote dispersion, they undergo significant molecular entanglement with anionic retarders. These findings validate fluorescence labeling as a robust tool for studying additive mechanisms in complex systems and suggest that competitive interactions between cationic polymers and anionic retarders necessitate precise dosage optimization to ensure slurry stability.

## Abstract 2

Fluorescent cationic polymer PDDN visualizes adsorption in cement slurries with anionic retarder interference, unlike TOC. Competitive cationic/anionic interactions govern dispersion, demanding optimized dosage in oil-well cementing.

## Graphical abstract

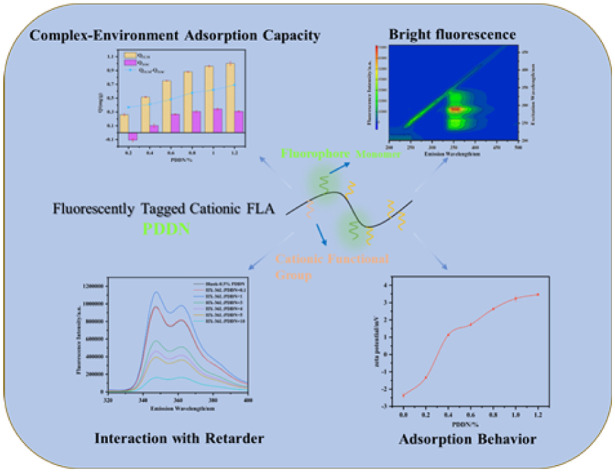

## 1. Introduction

Well cementing plays a pivotal role in ensuring wellbore integrity, zonal isolation, and long-term safe production.^1,2^ Fluid loss additives (FLAs) are essential for maintaining slurry mobility by preserving the aqueous phase content, thereby effectively minimizing fluid invasion and mitigating formation damage.^3–5^ However, with the continuous advancement of oil and gas exploration and the depletion of shallow resources, the industry focus has shifted towards developing deepwater and ultra-deep reservoirs (>6000 m) within complex geological formations.^6–8^ Nevertheless, these environments present significant technical challenges, particularly under high-temperature and high-pressure (HTHP) conditions.^9,10^ Therefore, elucidating the fundamental action mechanisms of FLAs is critical for the development of high-performance cement slurries capable of withstanding these rigorous conditions.

Concurrently, in the ongoing research of FLAs, the incorporation of cationic monomers to enhance salt tolerance and adsorption capabilities has attracted widespread attention.^11,12^ For instance, Bu *et al.* synthesized a novel amphoteric fluid loss additive using 2-acrylamido-2-methylpropanesulfonic acid (AMPS), acrylamide (AM), dimethyldiallylammonium chloride (DMDAAC), and itaconic acid (IA). Their study demonstrated that the synergistic effect between anions and cations effectively reduced the fluid loss volume to below 50 mL.^13^ Similarly, to address stability issues in high-salinity environments, Xia *et al.* developed a zwitterionic polymer composed of AMPS, DMDAAC, *N*,*N*-dimethylacrylamide (DMAA), and acrylic acid (AA). The results indicated that, compared to conventional additives, this polymer exhibited superior temperature and salt resistance while mitigating adverse effects on the compressive strength of the set cement.^14^ Furthermore, Yu *et al.* investigated the degradation mechanism of an amphoteric fluid loss additive containing methacryloyloxyethyl trimethyl ammonium chloride (DMC) at ultra-high temperatures. Their findings revealed that the failure of the additive under such conditions was primarily attributed to the detachment of side groups and the scission of the polymer main chain.^15^ Despite these advancements, research specifically focusing on the individual performance contribution of cationic moieties remains limited, particularly regarding their competitive interaction with commonly used anionic retarders. Moreover, regarding the investigation of adsorption mechanisms, traditional Total Organic Carbon (TOC) measurements struggle to circumvent interference from other organic admixtures.^16–18^ Consequently, there is a pressing need for a novel methodological approach to accurately investigate the adsorption behavior of fluid loss additives within such complex multi-component systems.

The functionality of fluorescent materials has expanded significantly from conventional roles in lighting and displays, with their use as tracers now established across varied domains such as biomedicine, environmental surveillance, and public safety.^19–22^ Accurate quantification of their fluorescence allows for the detailed tracking of target substance concentrations and associated physical and chemical transformations.

To address the challenges of quantifying adsorption in the presence of retarders and the lack of mechanistic insight into cationic polymers, this study utilizes precipitation polymerization to incorporate *N*-vinyl carbazole (NVC) into a cationic fluid loss additive, enabling fluorescent labeling to investigate the specific role of cationic moieties. The dosage of NVC was carefully optimized to achieve a critical balance between water solubility and fluorescence intensity. By determining the optimal excitation and emission wavelengths *via* fluorescence spectroscopy, a standard curve correlating fluorescence intensity with concentration was established. This method exhibits exceptional sensitivity and resistance to interference; crucially, unlike traditional Total Organic Carbon (TOC) analysis, it effectively circumvents the distorting effects of retarders, thereby facilitating the accurate investigation of adsorption behaviors in multi-component environments. Furthermore, by integrating fluorescence data with zeta potential analysis, particle size distribution measurements, and microscopic observations, this work systematically elucidates the impact of cations on slurry performance and the underlying adsorption mechanisms. Specifically, the study uncovers the competitive molecular entanglement between cationic additives and anionic retarders, offering valuable theoretical insights for the optimization of complex cement slurry formulations.

## 2. Experimental section

### 2.1. Materials and equipment

*N*,*N*-Dimethylacrylamide (DMAA, >98% purity), dimethyldiallylammonium chloride (DMDAAC, 60 wt% aqueous solution), and *N*-vinyl carbazole (NVC, >98% purity) were procured from J&K Chemical Ltd (Beijing, China). The initiators and pH regulators, including ammonium persulphate ((NH_4_)_2_S_2_O_8_, >99%), sodium bisulfite (NaHSO_3_, >99%), and sodium hydroxide (NaOH, >97%), were supplied by Sinopharm Chemical Reagent Co., Ltd (Shanghai, China). Class G oil well cement was provided by Jiahua Special Cement Co., Ltd (Sichuan, China). To prevent strength retrogression at elevated temperatures, silica flour (mineral high-temperature reinforcer) was obtained from the CNPC Engineering Technology Research Institute. Commercial additives, specifically the retarder (HX-36L, 25% active content) and anionic fluid loss additive (HX-12L), were acquired from Chengdu OMAX Petroleum Technology Co., Ltd (Sichuan, China).

### 2.2 Synthesis of poly(DMAA–DMDAAC–NVC)

The copolymer, designated as PDDN, was synthesized *via* precipitation polymerization employing a binary solvent system of ethanol and water (8 : 2 w/w).^23^ Initially, DMAA and DMDAAC were dissolved in the mixed solvent at a molar ratio of 9 : 1 to achieve a total monomer concentration of 20 wt%. The pH of the solution was subsequently adjusted to 6.0 using NaOH. Following this, *N*-vinylcarbazole (NVC) was introduced into the system at varying dosages ranging from 0.1 to 1.5 wt% relative to the total monomer mass.

The reaction mixture was transferred into a three-necked round-bottom flask fitted with a mechanical stirrer and a reflux condenser. To ensure an oxygen-free environment, the system was purged with nitrogen gas for 30 min. Polymerization was initiated by the addition of a redox initiator pair, (NH_4_)_2_S_2_O_8_ and NaHSO_3_ (1.0 wt% relative to monomers), and allowed to proceed at 45 °C for 6 h. Post-reaction, the resultant precipitate underwent purification through repeated washing with excess ethanol and filtration. The final product was dried in a vacuum oven at 50 °C until a constant weight was achieved.

### 2.3 Structural characterization

The chemical architecture of the synthesized copolymer was elucidated using Fourier Transform Infrared Spectroscopy (FTIR) and Proton Nuclear Magnetic Resonance (^1^H-NMR). FTIR spectra were recorded on an IRTRacer-100 spectrometer (Shimadzu, Japan) using the KBr pellet technique over a scanning range of 400–2000 cm^−1^ to identify characteristic functional groups. For ^1^H-NMR analysis, the polymer samples were dissolved in deuterium oxide (D_2_O), and spectra were acquired using an AVANCE III HD 400 MHz spectrometer (Bruker, Germany).

### 2.4 Performance evaluation of PDDN

#### 2.4.1 Cement slurry preparation

Cement slurries were prepared in strict accordance with API Recommended Practice 10B-2. The formulation was calculated based on the weight of dry cement (BWOC). Silica flour was incorporated at 50% BWOC to maintain the compressive strength of the set cement under high-temperature curing conditions. The retarder dosage was adjusted between 1% and 5% BWOC to ensure sufficient thickening time for conducting the API static filtration tests. Tap water was used as the mixing fluid at a ratio of 53% BWOC, while the dosage of the fluid loss additive ranged from 0.2% to 1.2% BWOC. All components were homogenized using a high-speed mixer prior to testing.

#### 2.4.2 High temperature API static filtration test

Filtration properties were evaluated following the API RP 10B-2 standard. The prepared slurry containing silica flour, retarder, and fluid loss additives was first conditioned in an ultra-high temperature pressurization curing simulator. The system was heated to the target temperature while the pressure was maintained between 70 and 110 MPa depending on the specific experimental requirements. After conditioning for the designated period, the slurry was cooled to below 90 °C. Subsequently, the slurry was transferred to a high-temperature and high-pressure stirrable fluid loss cell (BSRD-7071F, Liaoning Bassrett Petroleum Equipment Manufacturing Co., Ltd, China) to measure the fluid loss volume at the target temperature.

#### 2.4.3 Rheological measurements

The rheological behavior of the cement slurries was characterized using a ZNN-D6 six-speed rotational viscometer (Qingdao Haitongda Special Instrument Co. Ltd, China). The dial readings at different rotational speeds were recorded, and the rheological parameters, specifically the flow behavior index (*n*) and consistency coefficient (*k*), were derived from the data based on the Power Law model6.

### 2.5 Analysis of adsorption behavior

Zeta potential is commonly utilized to evaluate the adsorption and dispersion performance of fluid loss additives on cement particles.^24,25^ Generally, a higher absolute zeta potential value (|zeta|) facilitates the uniform dispersion of cement particles, which is conducive to the formation of a dense and impermeable filter cake during the filtration process. Zeta potential measurements were conducted using a Zeta Meter 3.0 (Zeta Meter Inc., USA). This instrument calculates the zeta potential by measuring the electrophoretic mobility of the particles in suspension.

The dispersion state of cement particles in the aqueous phase is a critical factor affecting the rheological properties and fluid loss performance of cement slurry.^26^ To visually and quantitatively elucidate the dispersion mechanism of PDDN, the particle size distribution and optical microstructure of the cement slurry were characterized. The particle size distribution of the cement slurry after aging was analyzed using a Baxter Bettersize 2000 Laser Particle Analyzer (Dandong, China) to assess the dispersion state. The dispersion morphology of cement particles was further observed using an ortho metallographic microscope (DM4 M, Leica, Germany).

The adsorption capacity of the polymer onto Class G oil well cement was quantified using the depletion method *via* two distinct techniques. First, the Total Organic Carbon (TOC) concentration in the filtrate was measured using a TOC-L CPH analyzer. Second, fluorescence spectroscopy was employed for more specific detection. Three-dimensional fluorescence spectra were obtained using an RF-6000 fluorescence spectrophotometer (Shimadzu, Japan) to determine the optimal excitation and emission wavelengths. A calibration curve was established using polymer solutions of known concentrations. The residual polymer concentration in the centrifuged filtrate was then determined based on fluorescence intensity, allowing for the calculation of the adsorbed amount.

## 3. Results and discussion

### 3.1 Synthesis optimization of poly(DMAA–DMDAAC–NVC)

**Fig. 1:**
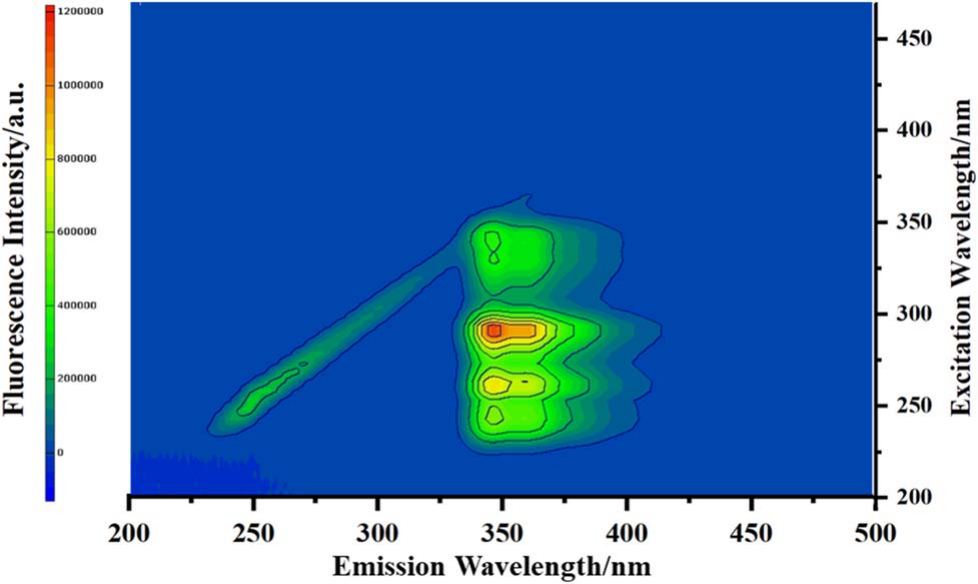
Three-dimensional fluorescence spectroscopy of PDDN.

The excitation and emission spectra of the synthesized poly(DMAA–DMDAAC–NVC) are presented in Fig. 1. As observed in the figure, the primary excitation region is concentrated between 275 and 300 nm. The maximum excitation wavelength was located at 290 nm, corresponding to a maximum emission peak at 347 nm. This indirectly confirms that NVC was effectively incorporated into the polymer.^27^

**Fig. 2:**
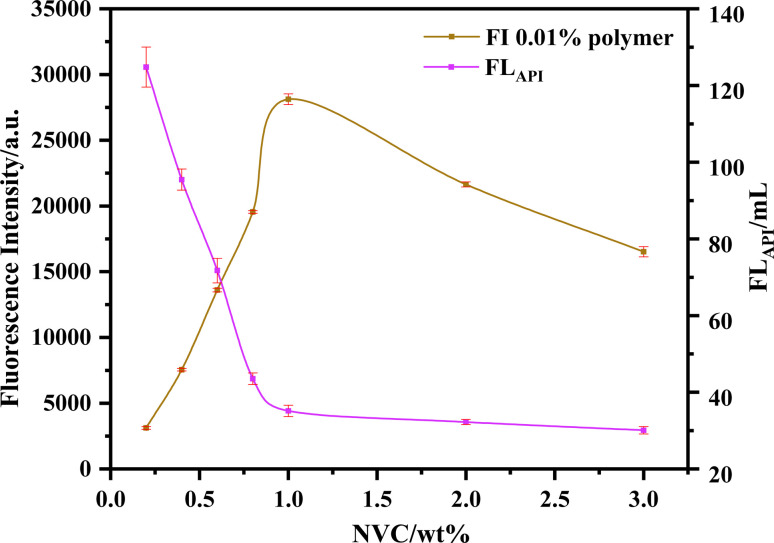
Effect of NVC dosage on fluorescence intensity and fluid loss (error bars represent standard deviation, *n* = 3).

However, due to the strong hydrophobicity of NVC, an excessive dosage significantly reduces the water solubility of the product, thereby affecting its performance. Conversely, an insufficient dosage compromises the effectiveness of precipitation polymerization and results in low fluorescence intensity. Therefore, the dosage of NVC (defined as the mass percentage of the total monomers) was optimized, with the results shown in Fig. 2.

With an increase in NVC dosage, the fluorescence intensity exhibited a trend of initial increase followed by a decrease. This is primarily because increasing the dosage introduces more fluorophores into the polymer, naturally enhancing fluorescence intensity. However, as the dosage increased further (with an inflection point at 1 wt%), the increased hydrophobicity of the polymer led to reduced solubility in water and subsequent precipitation, which resulted in a decrease in fluorescence intensity. In contrast, the fluid loss exhibited a gradually increasing trend with the increase in NVC dosage. This indicates that the introduction of NVC facilitates the polymerization process within this range, whereas higher molecular weight contributes to the reduction of fluid loss (Fig. 2).

Considering both the yield and fluorescence intensity, the optimal NVC dosage was determined to be 1 wt%, and the resulting product was designated as PDDN.

### 3.2 Characterization of PDDN

#### 3.2.1 The product structure was analyzed by FTIR and^1^H NMR

**Fig. 3:**
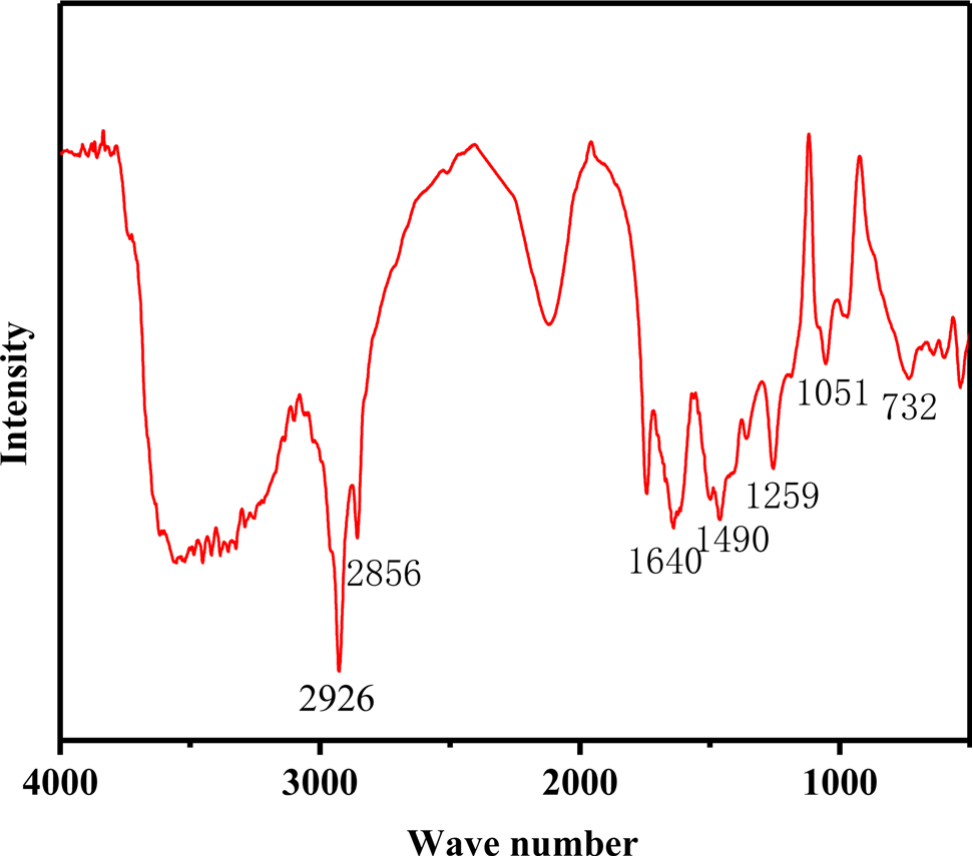
FTIR spectra of PDDN.

The FT-IR spectrum characterizing the structure of PDDN is presented in Fig. 3. The absorption bands at 2926 cm^−1^ and 2856 cm^−1^ are assigned to the stretching vibrations of –CH_2_ and –CH_3_ moieties. Regarding the characteristic peaks, the amide C=O stretching vibration of DMAA appears at 1640 cm^−1^.^28^ The bands at 1490 cm^−1^ and 732 cm^−1^ are ascribed to the skeletal vibration and out-of-plane bending of NVC, while the peaks at 1259 cm^−1^ and 1051 cm^−1^ are identified as the C–N stretching vibrations of DMAA and DMDAAC, respectively.^29^

**Fig. 4:**
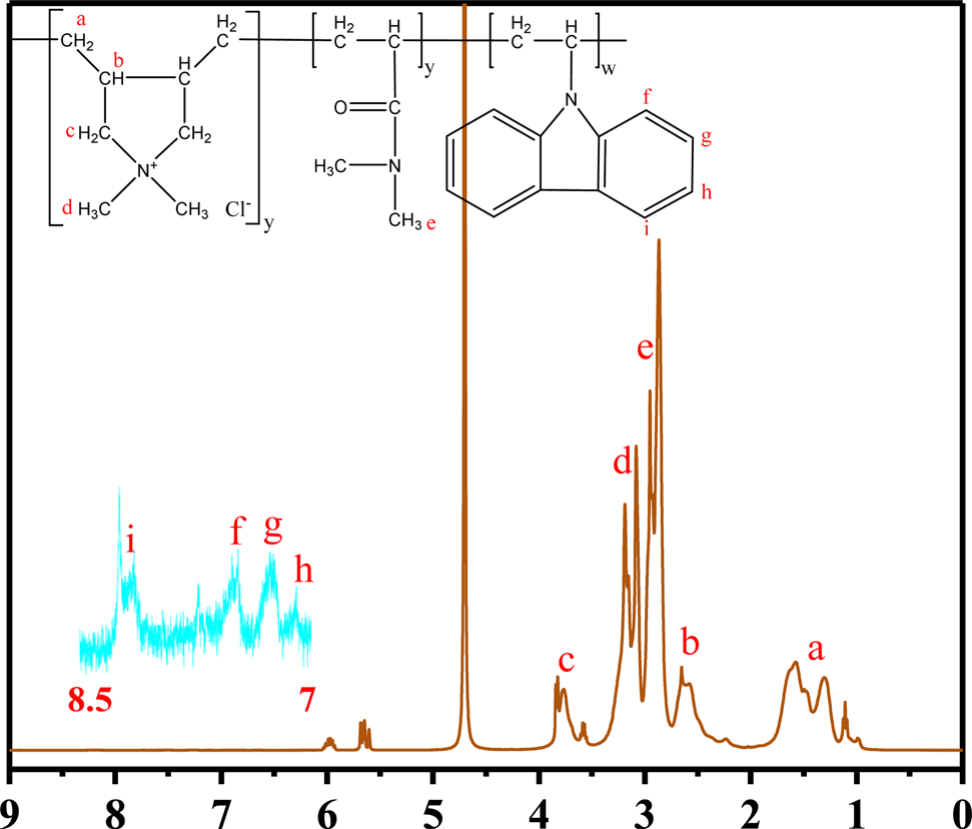
^1^H-NMR of PDDN.

The structure of PDDN was characterized using ^1^H-NMR spectroscopy, as illustrated in Fig. 4. All proton signals corresponding to distinct chemical environments are labeled a–i. The signals a (*δ* = 1.10–1.62 ppm) and b (*δ* = 2.58–2.63 ppm) are primarily attributed to the methylene (–CH_2_–) and methine (–CH–) protons on the polymer backbone. Signal c corresponds to the methylene protons (*N*–CH_2_–) within the cyclic structure of DMDAAC. Signals d *δ* = 3.18, 3.08 ppm) and e (*δ* = 2.95, 2.87 ppm) are assigned to the quaternary ammonium methyl groups (N^+^–CH_3_) of DMDAAC and the methyl groups (*N*–CH_3_) of DMAA, respectively. The aromatic protons derived from the –*N–*C=CH– group in the NVC monomer appear as multiple signals (f, g, h, and i) in the range of *δ* 7.00–8.50 ppm. In conjunction with the FTIR results, the ^1^H-NMR analysis confirms the successful synthesis of the target product.^30^

### 3.3 Adsorption standard curve

**Fig. 5:**
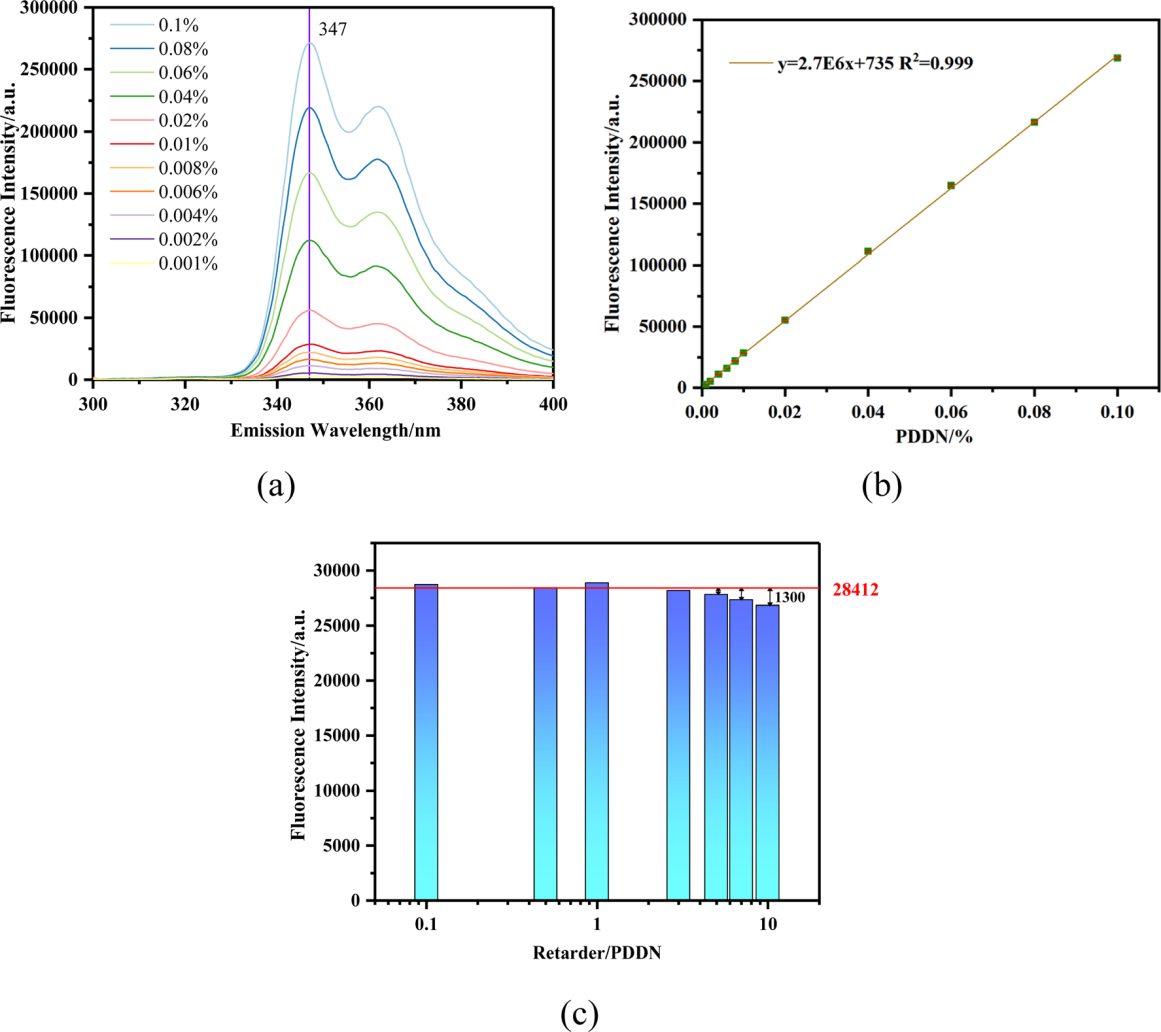
Adsorption standard curve:(a) emission spectra of PDDN at various concentrations (*λ*_ex_ = 290 nm); (b) standard concentration curve of PDDN (error bars represent standard deviation, *n* = 3) (c) effect of retarder dosage on fluorescence characteristics of PDDN.

In Section 3.1, the optimal excitation wavelength for PDDN was determined to be 290 nm. Consequently, to establish a standard calibration curve correlating PDDN concentration with fluorescence intensity, a series of polymer solutions with varying concentrations were measured. The results are presented in Fig. 5.

Fig. 5(a) illustrates the fluorescence emission spectra of the copolymer aqueous solutions. Upon excitation, the copolymer exhibited an emission profile characterized by fine structure, with a maximum emission peak located at 347 nm and a shoulder peak at 362 nm. These spectral features are highly consistent with the monomeric emission characteristics of isolated carbazole chromophores.^31^ Notably, no broad, structureless band was observed in the vicinity of 400 nm. Such broad bands are typically attributed to sandwich-like excimers formed between adjacent carbazole pendant groups, a phenomenon commonly observed in block copolymers or poly(*N*-vinylcarbazole) homopolymers. The absence of excimer emission confirms that the NVC units are randomly distributed along the polymer chain and are effectively isolated by the abundant DMAA and DMDAAC segments, thereby preventing the spatial aggregation of the hydrophobic fluorophores.^32^

Furthermore, the copolymer demonstrated excellent potential for quantitative detection. In the concentration range of 0.001% to 0.1%, the fluorescence intensity exhibited an exceptional linear relationship with the polymer concentration (*R*^2^ = 0.999), with a fitted equation of *y* = 2.7E6*x* + 735. The extremely high slope (2.7 × 10^6^) indicates the high sensitivity of this method, allowing for responsive feedback even to slight concentration changes. This characteristic effectively guarantees the reliable fluorescent tracking of the fluid loss additive. Meanwhile, the very low intercept (735) indicates minimal background interference.

In practical cementing operations, cement slurry filtrates invariably contain a variety of chemical additives, among which anionic retarders are ubiquitously employed. Given that the PDDN polymer chain incorporates cationic moieties (DMDAAC), there is a significant potential for electrostatic interactions with anionic retarders, leading to the formation of polyelectrolyte complexes. Theoretically, such structural alterations could compromise the fluorescence properties of the carbazole fluorophores. To evaluate the anti-interference performance of PDDN under these conditions, the variations in fluorescence intensity of Retarder/PDDN hybrid systems at various mass ratios were investigated. The results are presented in Fig. 5(c).

As illustrated in Fig. 5(c), the red reference line denotes the initial fluorescence intensity (28 412 a.u.) of 0.01% PDDN in the absence of the retarder. Experimental results indicate that the system exhibits exceptional resistance to interference at this concentration. Even in the presence of a high concentration of retarder (up to a mass ratio of 10 : 1), the fluorescence intensity of PDDN experienced only a marginal decline (retention rate >95%), with no significant fluorescence quenching observed. This stability can be attributed to the relatively low overall concentration of the solution, which ensures large intermolecular distances and minimizes strong intermolecular aggregation. Furthermore, the excellent water solubility of PDDN and the effective steric shielding provided by the macromolecular backbone protect the hydrophobic carbazole groups from the electrostatic interference of oppositely charged ions. These findings demonstrate that PDDN, acting as a fluorescent fluid loss additive, effectively guarantees reliable performance for subsequent fluorescence tracking applications.

### 3.4 Performance evaluation of PDDN

#### 3.4.1 Evaluation of rheological properties

**Fig. 6:**
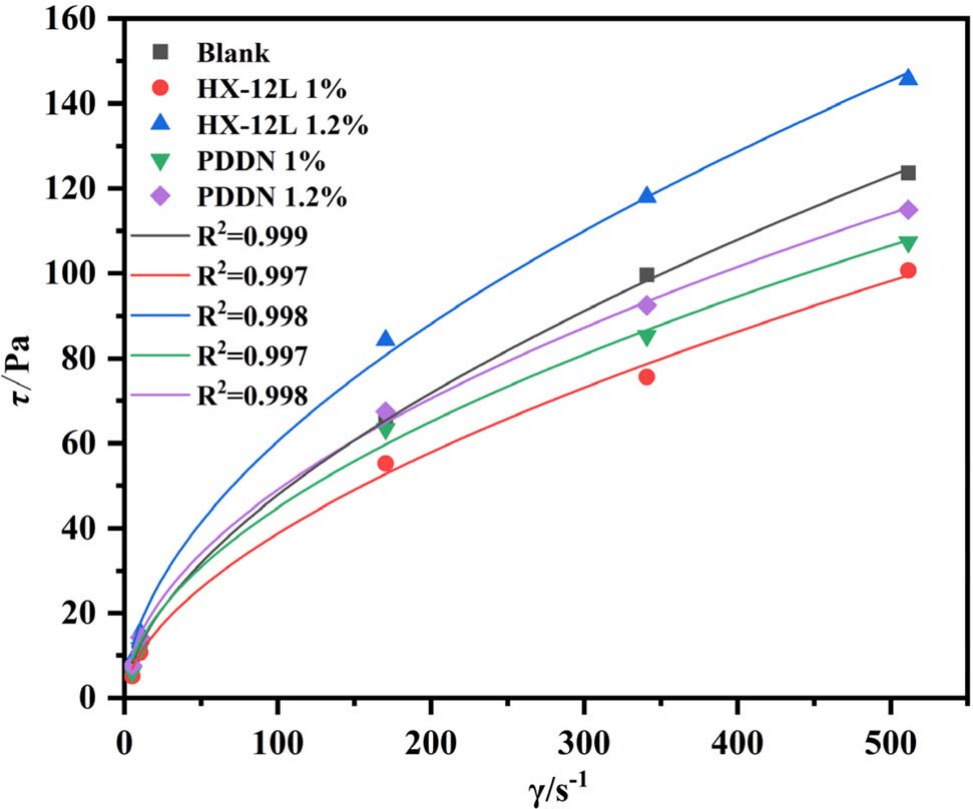
Power-law model fitting for rheological properties of cement slurry.

The influence of distinct fluid loss additives on the rheological behavior of cement slurries was investigated, with the corresponding results presented in Table 1. As illustrated in Fig. 6, which depicts the rheological curves (shear stress *vs.* shear rate) for slurries treated with varying dosages of HX-12L and PDDN at 25 °C, the solid lines denote the fitting results derived from the Power Law model. All tested cement slurries exhibited characteristic non-Newtonian fluid behavior and demonstrated a high degree of fit with the Power Law model, as evidenced by correlation coefficients (*R*^2^) exceeding 0.99.^33,34^ Meanwhile, compared to the blank sample, the addition of HX-12L and PDDN at a dosage of 1% can both reduce the viscosity of the cement slurry and improve the rheological properties of the cement slurry, and the dispersion performance of HX-12L is better. However, at a dosage of 1.2%, the viscosity of the slurry with HX-12L begins to rise rapidly. This is mainly because the anionic fluid loss additive can adsorb well onto the surface of cement particles, increasing the electrostatic repulsion between particles and promoting cement dispersion, thereby improving the fluidity of the cement slurry at low dosages; however, with the increase of dosage, the polymers in the solution begin to entangle with each other and form a spatial network structure with the adsorbed cement particles, leading to an increase in viscosity. Relatively speaking, the cationic type PDDN has poorer dispersion performance for cement particles, but at high dosages, it is still able to maintain good fluidity. This may be attributed to its suitable adsorption properties and larger steric hindrance, thereby avoiding the rapid formation of a spatial network structure that leads to a rapid rise in viscosity.

**Table 1:** Measurement of rheological properties of cement slurry

Sample	Dosage/%	Temperature/°C	Test value					Rheological parameters	
			*φ*300	*φ*200	*φ*100	*φ*6	*φ*3	*n*	*K*
Blank	0	25	242	195	128	24	14	0.587	3.19
HX-12L	1	25	197	148	108	21	10	0.577	2.71
	1.2	25	285	231	165	30	17	0.545	4.89
PDDN	1	25	210	167	124	23	12	0.538	3.76
	1.2	25	225	181	132	28	15	0.524	4.37

#### 3.4.2 Compressive strength test

**Fig. 7:**
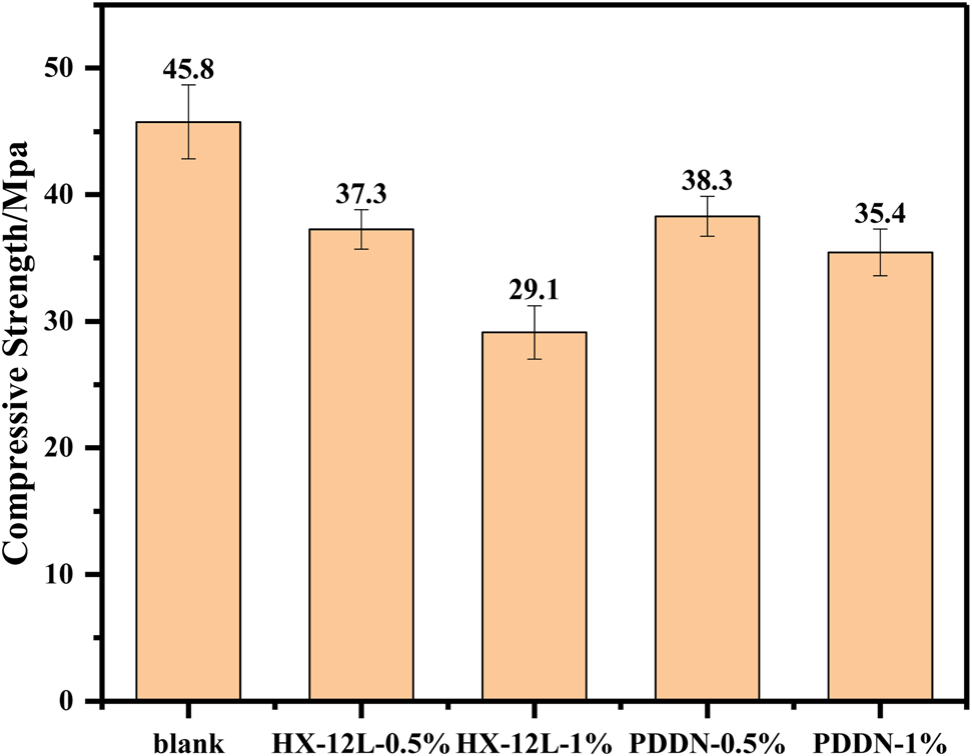
Effect of different fluid loss additives on the compressive strength of cement stone(24 h curing) (error bars represent standard deviation, *n* = 3).

The introduction of fluid loss additives (FLAs) into cement slurry systems often leads to a reduction in the compressive strength of hardened cement.^35–37^ Therefore, a study was conducted to investigate the effects of two types of FLAs on the compressive strength of cement stone, with the results presented in Fig. 7. As shown in the figure, the addition of FLAs resulted in a noticeable decrease in compressive strength compared to the blank sample, and the extent of reduction increased with higher FLA concentrations. However, compared with the anionic fluid loss additive, PDDN exhibited a relatively smaller decline in strength. This difference is primarily due to the strong adsorption capacity of anionic FLAs and their functional groups, which are often similar to those of retarders. These characteristics make them more likely to interfere with the cement hydration process, thereby slowing the strength development of the cement stone. In contrast, cationic FLAs possess weaker adsorption capacity and exert less influence on the hydration process, which mitigates their negative impact on the strength of hardened cement.

#### 3.4.3 Evaluation of filtration control performance

**Fig. 8:**
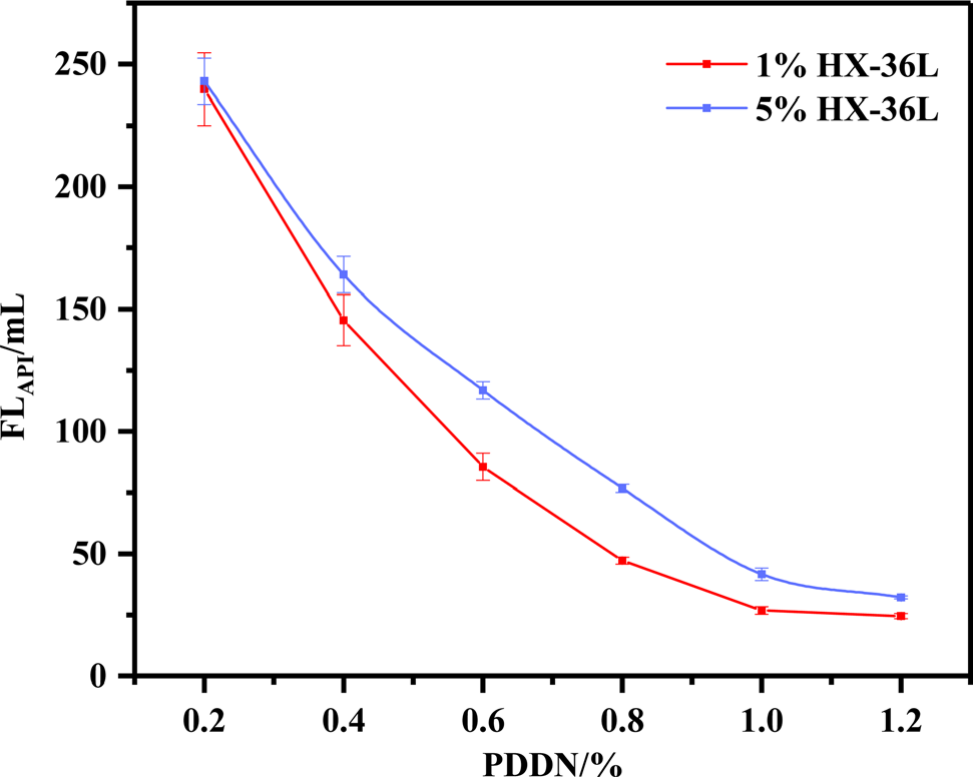
Relationship between PDDN dosage and fluid loss (90 °C) (error bars represent standard deviation, *n* = 3).

The performance of PDDN as a fluid loss additive was evaluated to provide a macroscopic basis for the subsequent investigation of cationic adsorption behavior, with the results illustrated in Fig. 8. A comparative analysis reveals that the fluid loss decreased rapidly with increasing PDDN dosage and stabilized after reaching a dosage of 1%. This stabilization is likely attributable to the saturation of the additive adsorbed onto the surface of the cement particles.

Simultaneously, the dosage of the retarder exerted a certain influence on the fluid loss. Although the adsorption sites for the two additives differ, the retarder likely entangled with the fluid loss additive, inducing a polyelectrolyte complexation reaction. This interaction consumed a fraction of the effective PDDN molecules, consequently reducing their adsorption onto the cement particles. However, the overall decline in performance was minimal, which can be primarily ascribed to the significant steric hindrance of PDDN that effectively resisted anionic interference to a certain extent.

**Fig. 9:**
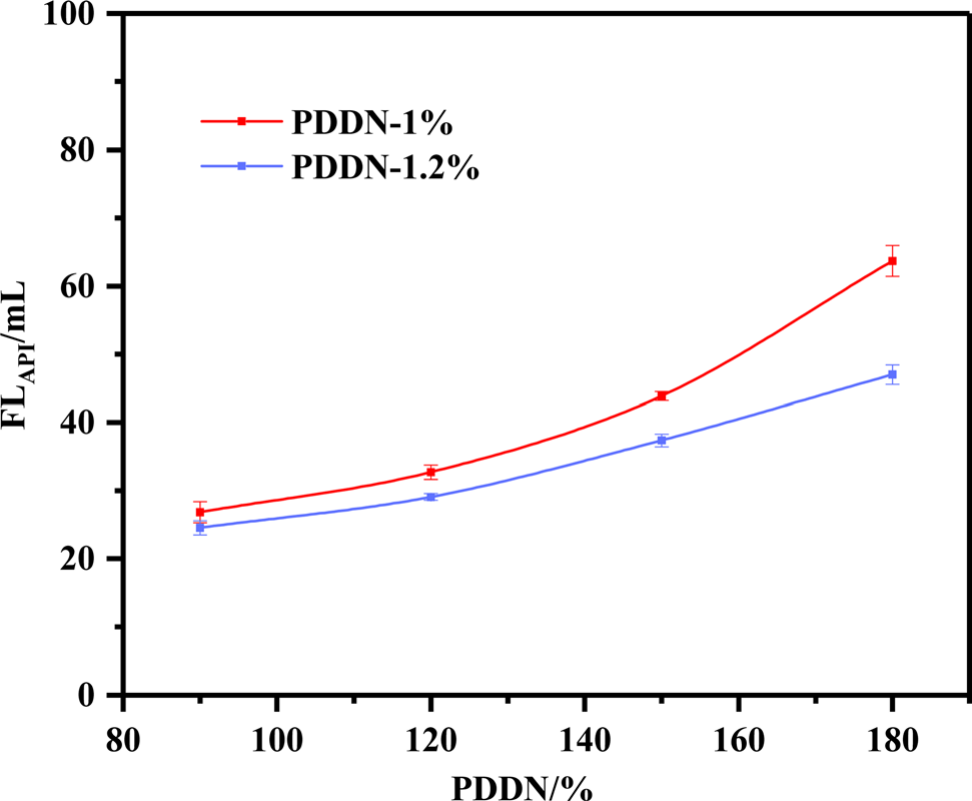
Effect of temperature on fluid loss of PDDN-containing slurry (error bars represent standard deviation, *n* = 3).

Furthermore, the impact of temperature on PDDN performance was investigated, as depicted in Fig. 9. The results demonstrate that fluid loss gradually increased as the temperature was elevated from 90 °C to 180 °C. This phenomenon arises because high temperatures intensify molecular thermal motion, potentially leading to the coiling of polymer chains or their partial desorption from the cement particle surface. Notably, when the dosage was increased to 1.2%, the system maintained favorable fluid loss control. This stability is likely due to the presence of rigid cyclic monomers within the molecular structure, which restricted the conformational changes of the polymer chains at elevated temperatures and mitigated thermal degradation.

### 3.5 Study on PDDN adsorption behavior

#### 3.5.1 Methods employed to assess adsorption capacity

Accurate quantification of polymer adsorption on cement particles is critical for elucidating the working mechanism of fluid loss additives. However, in practical cementing formulations, the presence of other organic admixtures, such as retarders, often complicates traditional analysis. To address this, the adsorption isotherms of PDDN were evaluated using both Total Organic Carbon (*Q*_TOC_) and the fluorescence labeling method (*Q*_FLM_) under varying retarder dosages.

**Fig. 10:**
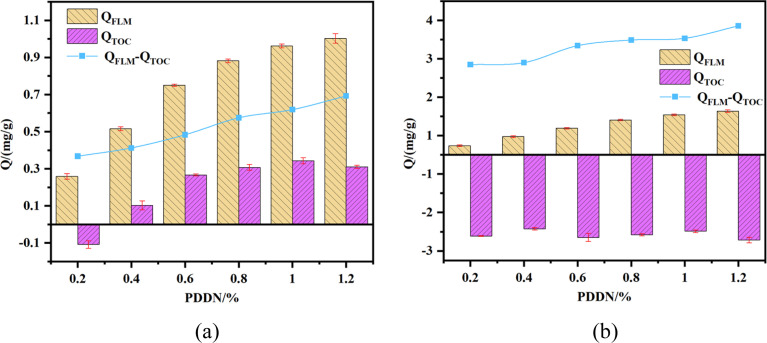
Adsorption amount (measured by TOC and FLM) as a function of PDDN dosage (a) is at low retarder concentration; (b) is at high retarder concentration (error bars represent standard deviation, *n* = 3).

As illustrated in Fig. 10, a significant discrepancy exists between the values obtained from the two methods. In the presence of the anionic retarder, the *Q*_TOC_ values were consistently suppressed. Notably, at high retarder dosages (Fig. 13), *Q*_TOC_ exhibited anomalous negative values (approx. −2.5 mg g^−1^) across the entire PDDN concentration range. This phenomenon is attributed to the non-selective nature of TOC analysis, where the high background organic carbon concentration from the retarder in the bulk solution masks the relatively smaller variations caused by PDDN adsorption, rendering the method unreliable for multi-component systems. In contrast, the *Q*_FLM_ results demonstrated a logical Langmuir-like adsorption behavior, with adsorption capacity increasing with PDDN dosage. Fluorescence labeling tracking enables effective measurement of the adsorption capacity of fluid loss reducers even in the presence of retarder interference. Nevertheless, the adsorption of PDDN remains influenced to some extent by anionic retarders, exhibiting a decrease with increasing retarder dosage. Simultaneously, the relatively high *Q*_FLM_ values confirm that PDDN retains significant adsorption capability onto cement hydration products, despite the potential electrostatic complexation with the anionic retarder. Consequently, this study establishes the fluorescence labeling method as an effective approach for investigating the adsorption thermodynamics of such fluid loss additives.

#### 3.5.2 Intermolecular interactions between PDDN and anionic retarder

**Fig. 11:**
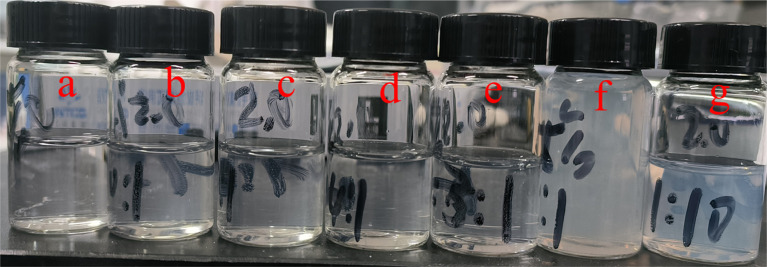
Blended solutions of HX-36L and PDDN at different concentration ratios. Sample (a) is the blank containing 0.5% PDDN; samples (b) through (g) correspond to HX-36L : PDDN ratios from 10 to 0.1.

Since the introduction of cations into fluid loss additives often leads to interactions with anionic retarders, the incorporation of fluorophores can effectively reflect the progress of such interactions.^38,39^ Therefore, to deeply investigate the compatibility and interaction mechanism between the cationic polymer PDDN and the anionic retarder (HX-36L), blended solutions with varying mass ratios (HX-36L : PDDN ranging from 10 : 1 to 0.1 : 1) were prepared, and their macroscopic states were observed. As shown in Fig. 11, the appearance of the mixed solutions exhibited a distinct gradient transition with changes in the ratio. At specific high ratios (a–d), the solutions maintained a transparent and homogeneous state. This indicates that within this range, the two components formed soluble polyelectrolyte complexes, or the excess of one component provided sufficient residual charge to maintain the good dispersion and solvation of polymer chains in the aqueous phase.^40^ As the mixing ratio changed (e–g), the turbidity of the solution gradually increased, eventually transforming into a milky, opaque suspension. This is primarily because when the mixing ratio approached the isoelectric point of the system, effective neutralization of positive and negative charges occurred. According to the classical theory of polyelectrolyte complexation, this charge neutralization causes the polymer chains to undergo a conformational transition from extended coils to collapsed globules, leading to the formation of micron-sized coacervates or aggregates.^40^

**Fig. 12:**
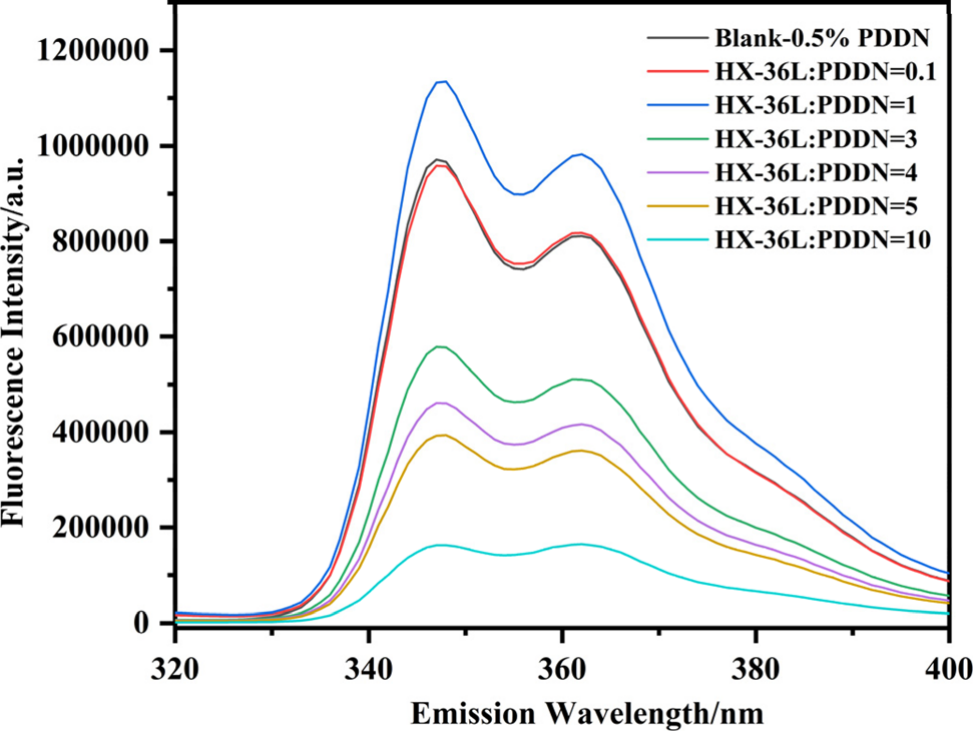
Variation in fluorescence intensity with different HX-36L-to-PDDN ratios.

Simultaneously, the fluorescence intensity of the corresponding solutions was analyzed, with the results presented in Fig. 12. When the dosage of the retarder was low, the overall fluorescence intensity of the sample showed no significant attenuation. However, with an increase in retarder dosage, the fluorescence intensity began to decline rapidly. Interestingly, the fluorescence intensity exhibited a trend inverse to the turbidity: the transparent samples with high retarder dosages (b–d) showed the most significant quenching. This seemingly counter-intuitive phenomenon—quenching in the absence of macroscopic precipitation—provides deep molecular insight. It suggests that within the soluble complexes, the PDDN chains are tightly wrapped by retarder molecules. In the high-retarder regime: although no macroscopic precipitate forms, the excess retarder molecules tightly surround the PDDN chains *via* strong electrostatic attraction. This creates a “cage effect,” causing the PDDN coils to undergo a microscopic collapse into dense, soluble globules. Within these globules, the NVC fluorophores are forced into close proximity, inducing strong static quenching or π-π stacking.^41^ In the low-retarder regime: while macroscopic aggregates form due to bridging, the lower density of retarder molecules allows parts of the PDDN chain to retain a more extended conformation within the loose flocs, thereby preserving higher fluorescence intensity.

Therefore, this experiment confirms that a distinct “compatibility window” exists when PDDN is used in conjunction with anionic retarders. Within this window (ratios of 1 : 1–1 : 3), PDDN maintains a dissolved state and exhibits relatively weak mutual interference with the retarder.

#### 3.5.3 Dispersion mechanism analysis

**Fig. 13:**
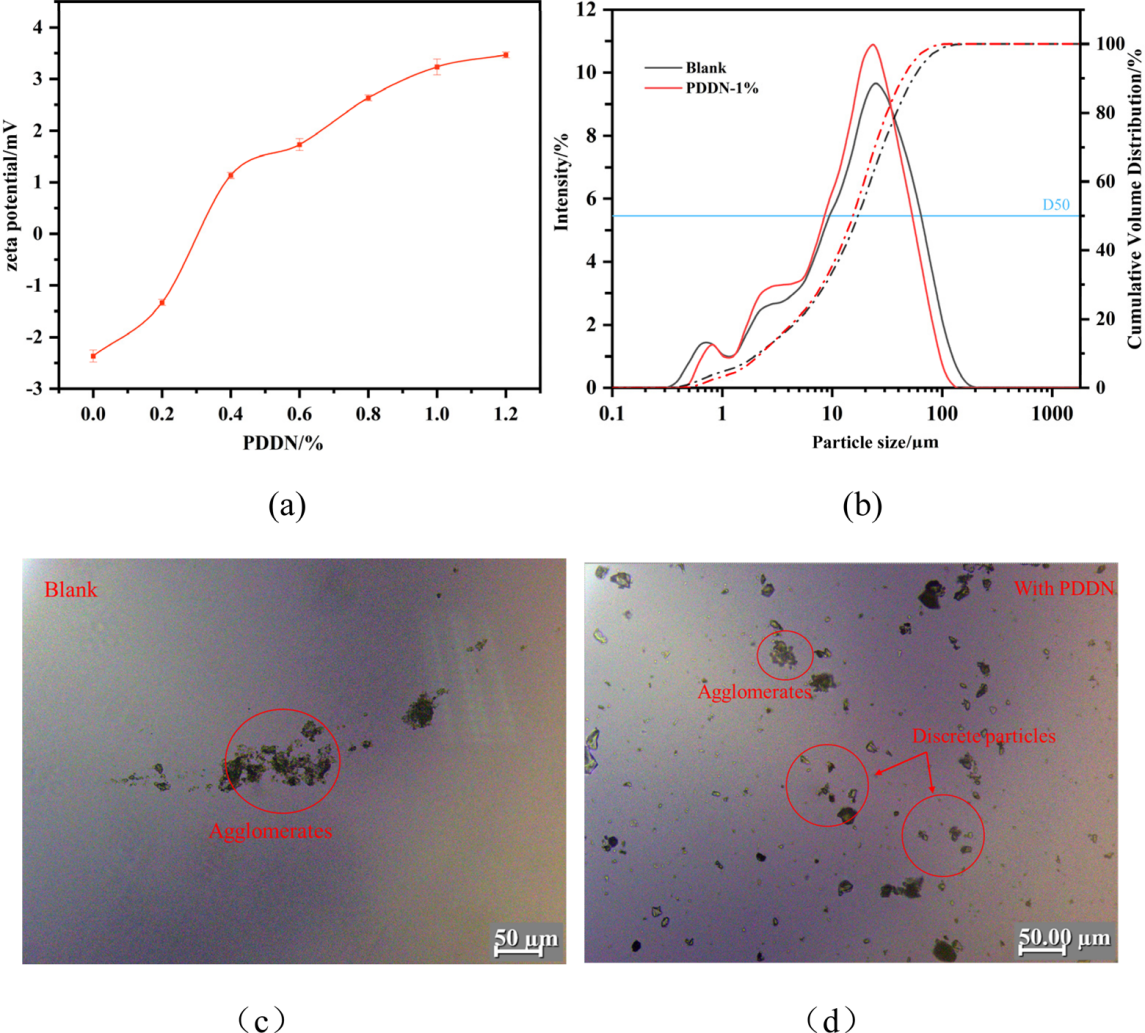
Mechanism of PDDN improving the dispersion of cement slurries: (a) zeta potential of cement particles as a function of PDDN dosage (error bars represent standard deviation, *n* = 3); (b) particle size distribution of cement slurries with and without PDDN; (c) image of cement slurry magnified by 200 without PDDN; and (d) microscopic image of cement slurry with 1.0% PDDN.

The influence of PDDN dosage on the zeta potential of the cement slurry was investigated, with the results illustrated in Fig. 13(a). As observed in the figure, with an increase in PDDN dosage, the zeta potential gradually shifted from an initial negative value to a positive value. This charge reversal indicates that the cationic groups of PDDN effectively adsorbed onto the surface of the cement particles, leading to a progressive accumulation of positive surface charges. However, as the dosage was further increased, the rate of increase in zeta potential diminished and eventually reached a plateau. This stabilization is primarily attributed to the saturation of adsorption on the cement surface; consequently, further addition of the polymer could not significantly enhance the zeta potential. This finding aligns well with the previously measured adsorption capacity data. Nevertheless, the final absolute zeta potential (|zeta|) remained relatively low, suggesting that while the cationic functional groups successfully adsorbed onto the cement particles to a certain extent, their contribution to enhancing the dispersion capability of the cement particles was limited.

The particle size distributions of the blank cement and the sample with a 1% PDDN dosage are compared in Fig. 13(b) (The average of three measurements). As can be seen from the figure, the distribution curve of the blank sample displays a broad main peak centered at 20–30 µm, with a distinct shoulder peak in the range of 100 µm. This multimodal distribution indicates severe flocculation and the presence of large agglomerates formed between cement particles due to hydration and electrostatic attraction. Conversely, the addition of PDDN resulted in a significant shift of the distribution curve to the left. The intensity of the main peak increased significantly, while the content corresponding to coarse particles (>50 µm) decreased. This shift towards a smaller particle size range implies that larger flocculated structures were effectively broken down into finer individual particles. The decrease in the median particle diameter (D50) further confirmed that PDDN prevents particle agglomeration. Moreover, this indicates that the cationic fluid loss additive can effectively promote the dispersion of cement particles and reduce the particle size of cement particles. Combined with the observations from high-magnification microscopy, as shown in Fig. 13(c) and (d), obvious agglomeration of cement particles occurs in the blank cement sample, which leads to the formation of a loose packing state during the accumulation process of filter cake formation. However, after the introduction of PDDN, the situation was improved; the agglomerates became fewer and smaller but did not completely disappear. This further indicates that the cations can adsorb onto the surface of cement particles to a certain extent, enhancing the mutual repulsion between particles, but the enhanced repulsion ability is limited.

#### 3.5.4 Comparison with traditional analytical methods

To assess the practical utility of the fluorescence labeling method (FLM), it was compared with traditional total organic carbon (TOC) analysis. Although TOC analysis is widely employed, it lacks analytic specificity in multi-component formulations; consequently, previous researchers have primarily measured adsorption only when fluid loss additives were introduced individually.^11,17,18^ As evidenced by our data (Fig. 13), a high organic retarder background caused TOC measurements to yield anomalous negative adsorption values, confirming the unreliability of this method in complex fluids. In contrast, FLM maintains high specificity, enabling the accurate quantification of PDDN adsorption even under severe interference.

Operationally, FLM can generally be more intricate. While both techniques analyze the filtrate following centrifugation and dilution, FLM necessitates the prior incorporation of a fluorophore and the establishment of a calibration curve to acquire adsorption data. However, in the presence of interfering agents, TOC analysis becomes significantly more complex, requiring time-consuming pre-separation steps (*e.g.*, dialysis) to eliminate the interferents. The primary limitation of the fluorescence labeling approach is the prerequisite to synthesize fluorescently labeled polymers, whereas TOC analysis is universally applicable to any organic additive without structural modification. Furthermore, potential fluorescence quenching effects induced by the external environment must be carefully calibrated and corrected for, although our linear standard curves indicate that these effects remain manageable within typical dosage ranges. Nevertheless, for investigating the complex interaction mechanisms inherent in modern cementing systems, the specificity of FLM provides critical solutions and insights that traditional methods cannot offer.

## 4. Conclusion

(1).Fluorescent marker groups were successfully incorporated into the cationic fluid loss additive *via* precipitation polymerization, endowing it with excellent fluorescence properties. The maximum excitation wavelength is 290 nm and the emission wavelength is 347 nm, indicating that the NVC units are randomly distributed along the polymer chain and are effectively isolated by a large number of DMAA and DMDAAC segments. Furthermore, the additive retains excellent fluid loss control performance.

(2).By measuring the fluorescence intensity of PDDN at various concentrations, a robust standard curve for fluorescence intensity was established (*y* = 2.7E6*x* + 735), demonstrating exceptional sensitivity. Within the measurement range, PDDN exhibits strong resistance to interference from retarders, maintaining its stable fluorescence characteristics.

(3). Performance evaluation of PDDN reveals that the introduction of cationic groups can, to a certain extent, improve the rheological properties of the cement slurry, mitigate the adverse impact of the fluid loss additive on cement strength, and enhance fluid loss control. However, its ability to disperse cement particles is inferior to that of anionic additives.

(4). Compared with the traditional TOC method, the fluorescence labeling method enables the accurate measurement of the adsorption capacity of the fluid loss additive even in the presence of retarders. While the discrepancy between the two methods is minimal at low retarder dosages, the deviation increases significantly with increasing retarder dosage.

(5).Combined analyses of fluorescence labeling, zeta potential, and particle size distribution reveal that the cationic groups in the fluid loss additive undergo significant entanglement with anionic retarders, necessitating the careful regulation of their respective dosages. Additionally, while the cationic groups possess the ability to improve the zeta potential and particle size distribution of cement particles to a certain degree, this capacity is limited.

## Conflicts of interest

The authors declare that they have no known competing financial interests or personal relationships that could have appeared to influence the work reported in this paper.

## Data Availability

All data supporting this study are available within the article.
